# The Odontological Society of Great Britain

**Published:** 1885-05

**Authors:** 


ARTICLE II.

### THE ODONTOLOGICAL SOCIETY OF GREAT BRITAIN.

The Ordinary Monthly Meeting of this Society was held on May 4th, Mr. Richard White, L. D. S. Eng,, Vice-President in the Chair.

Mr. E. Lloyd Williams exhibited a model of the upper jaw of a lady, aged sixty, showing a large mass of hypertrophied gum tissue attached to the palate by a large flat pedicle. The occurrence of such growths in connection with the wearing of plates was not uncommon and this one was remarkable only on account of its size. The patient had worn a plate, retained by springs, for fifteen years.

Mr. Charters White remarked that such growths were often met with in patients who had been wearing for some time a misfitting plate. In this case the edge of the plate had evidently cut into and irritated the mucous membrane of the palate. He thought the best plan would be to make a plate to cover the whole of the palate, the pressure of which would soon cause the disappearance of the mass.

Mr. Browne-Mason (Exeter) exhibited models of the mouth of a girl, aged eleven, showing a remarkable malformation of the jaws. The upper jaw receded so that the incisors closed a quarter of an ineh within the lower, and the only teeth which articulated properly were the first permanent molars on the right side. He should be very glad to receive any suggestions as to treatment, though he feared the case was incurable.

He also handed round a model which he had received from Mr. Parkinson, of Bath, showing unusually large and well-formed supernumerary laterals.

Mr. W. A. Hunt (Yeovil) read the following notes of an instructive case of neuralgia coincident with the advent of the wisdom teeth:—

"A lady vocalist, some three years ago, suffered greatly from ordinary neuralgic pains on the right side of the head, affecting all three divisions of the fifth nerve, with pain apparently located in the first right lower molar. An irregular practitioner extracted this tooth, which he said was sound, but the patient experienced little benefit. Under the hands of her medical attendant, after a six month's course of quinine, &c., the pain did, however, slowly abate, but only to return as badly as ever on the *left* side.

"She then consulted me, but, she said, with little hope of obtaining relief. Her age was twenty-eight, and her general appearance healthy, but the pain made her miserable. Her teeth were all well formed and regular, but seemed firmly compressed against each other. The right lower wisdom tooth only had been erupted, and on this side the first lower permanent molar had been extracted, as already mentioned.

"The advancing wisdom tooth must, I thought, have been the cause of her pain, and had the second molar been extracted instead of the first, relief would have followed almost at once, instead of taking nearly six months, and the expenditure of so much medical skill and quinine. I therefore examined the left side carefully, and by passing a fine probe through a little dimple in the gum, I felt the wisdom tooth beneath. I at once removed the second molar under gas; some relief was at once experienced. I advised her to discontinne all medical treatment, and in two or three days the pain entirely subsided.

"A year elapsed, and she again consulted me with the same kind of symptoms on the *right* side. Here I could see a presenting cusp of the upper wisdom tooth. I extracted the second molar and relief again followed.

"In six months' time she again presented herself with the same typical pain on the *left* side, but more severe than ever. The most careful and prolonged examination failed to disclose the slightest sign of the advent of a wisdom tooth, or even of its existence; yet from former experience, I, without hesitation, extracted the second molar under gas, and I have here the tooth for your inspection. The specimen clearly shows the injury done by the advancing, though buried, tooth; the distal half of the posterior buccal root and some of the crown have disappeared, and the nerve canal is completely exposed for more than half the length of the root. It was impossible to diagnose this till after the extraction. The operation was followed by immediate relief, and the patient is now absolutely free from neuralgia.

"I may observe that no signs of local iflammation or irritation were discoverable from first to last in this case. The treatment adopted of extracting a useful second molar instead of digging out the less useful third molar was, I believe, correct in this case. All four wisdom teeth were large, well-developed healthy teeth, and eventually took up excellent and useful positions.

"The case is instructive, as its history is so definite and complete, and strikingly illustrates cause and effect no less than four consecutive times in the same individual; and lastly, as illustrating the serious and unsuspected damage that a buried tooth can inflict by pressure against the roots of a neighbor which stands in the way of its progress."

The Chairman said Mr. Hunt's case, or rather series of cases, was exceedingly interesting. He thought that trouble of this sort from the wisdom teeth was more common than it was generally considered to be. At all events, he met with such cases pretty frequently in his own practice, and had almost come to look upon wisdom teeth as natural enemies, to be got rid of whenever an opportunity offered. Recently his son, Mr. Wentworth White, had asked his advice with regard to a gentleman, aged forty, who had for some time been suffering severely from neuralgia affecting the left side of the face. He had consulted another practitioner, who had extracted the second upper bicuspid, which was found to be sound, and this gave no relief. Mr.

White could find nothing wrong with the teeth on that side in the lower jaw, but on carefully testing the upper teeth, the second molar was found to be sensitive, and it was decided to extract it. It was then found that the pressure of the wisdom tooth had made a cavity on the distal surface. The patient obtained immediate relief.

Mr. J. S. Turner thought that most of those present must be familiar with such cases. The wisdom tooth, however, was not the only tooth which was capable of causing mischief of this sort. At the March meeting, Mr. White showed a lateral which had undergone very extensive absorption, owing to the pressure of a neighbouring tooth, and quite recently he had himself removed a lateral in in exactly the same condition, absorption having taken place to such an extent as to expose the pulp cavity, due to the pressure of the canine, which was coming down in front of it. A remarkable feature of the case was that the patient, a youth, said it had not caused him any inconvenience.

Mr. C. J. B. Wallis showed the skull of a Zulu ; a very fine hippopotamus skull; the jaws of a sword fish (also a very fine specimen) ; and the jaws of a large extinct animal, which he believed to have been an icthyosaurus, and had been dug up in Egypt.

Mr. Hutchinson remarked that the hippopotamus skull was a magnificent specimen, and begged Mr. Wallis to use his influence with the owner to induce him to present it to the Society. The skull then in the Museum was not nearly such a fine one.

Mr. D. Hepburn showed a model of the mouth and jaws of a young man, aged twenty-three, who had been treated at St. George's Hospital thirteen years ago for "fever." He had at the same time some necrosis of the jaw, and extensive sloughing of the soft parts. Cicatrization eventually took place, but accompanied by anchylosis of the lower jaw. The jaw was now practically immovable; he had not the slightest power of mastication, and only a very small aperture between the teeth on the left side of the

2

mouth. Contraction seemed to be still going on, for the upper teeth were being slowly forced outwards. In spite of his inability to masticate, the patient enjoyed very good health, and Mr. Hepburn did not feel justified in advising any operative interference at present.

Mr. R. H. Woodhouse showed a model of the upper jaw of a man, aged thirty-four, which had been sent to him by Dr. Walker. At the age of ten the right permanent central was broken at the cervical line by a blow from a stone. Abscess ensued, and the root was extracted three or four months after the accident. Three years ago, seventeen years after the accident, the right permanent canine showed signs of eruption. The permanent right lateral and temporary canine were firmly articulated.

He also handed round a model of the upper jaw of a young man, aged seventeen, which had been sent as a donation to the Museum by Mr. Adams Parker, of Birmingham. His first dentition was perfectly natural. With the second dentition a supernumerary tooth appeared to the right of the middle line; this became loose and was extracted, when a second supernumerary tooth appeared in the same position. It was firmly implanted and quite sound.

The Chairman then called upon Dr. St. George Elliott for his communication on "Bridge-work."

Dr. Elliott said the subject of his communication was one which had not as yet been often discussed at meetings like the present. Indeed it was comparatively a new subject. For although bridge-work was actually a new invention—he had himself seen fifteen years ago a good example of this kind of work in the mouth of a patient, and it had been in use fifteen years at the time he saw it—yet it was only during the last five or six years that it had come into anything like common use in the profession. Like other methods it might be carried to an extreme, and used without judgment in cases were a plate would have answered much better; still it was very useful in suitable cases. Patients were sometimes met with who had a very strong objection to wearing a plate. Singers, for instance, found a plate very inconvenient; very nervous and irritable patients also not unfrequently objected to them. In such cases "Bridging" often afforded a satisfactory means of remedying defects.

It was sometimes asserted that bridge-work was likely to cause trouble, and do harm to the teeth which served as supports, owing to the difficulty of preventing accumulation of food about the parts; but this was a mistake. The work might and ought to be done in such a manner that no inconvenience whatever should arise from this cause, and perfect cleanliness could be maintained by the patient with less trouble than where a plate was worn.

He had prepared some diagrams which would illustrate some of the conditions under which this method of treatment might be beneficial. The first case he would mention was that of a German baron, who was first treated on this plan about five years ago by a dentist of Dresden. In this case the right upper lateral and canine had been fastened to the first bicuspid aud central. The bar was attached at one end of the lingual aspect of the central by a gutta-percha filling, and was anchored at the other end to the bicuspid by a gold filling. It was evident that a considerable strain fell upon the central, and it might have been supposed that the gutta-percha stopping would have given way, but it did not. The work lasted two years, and then the lateral broke off from the bar. The patient came to Dr. Elliott in order tc have the breakage repaired; he expressed himself as highly satisfied with what had been done, declaring that he had never had any comfort from artifical teeth until he had this bridge in place of a plate which he had previously been wearing. Dr. Elliott removed the piece with some difficulty, replaced the lateral, and refastened it as before to the central, only using gold instead of gutta-percha. This time it only lasted six months, when the central, to which the bar was attached, broke off. Dr. Elliott then cut down the stump of the central to the level of the gum, and attached to it a porcelain crown with a gold backing by means of a screw pin and nut. One end of the bridge was then soldered to the gold backing, and the other carried to the bicuspid and securely anchored to it by amalgam. This had lasted well, and had given great satisfaction to the patient.

Some amount of judgment and experience was required in adapting this method to particular cases. As an illustration of this he would mention a case which had come under his notice. The patient had lost his right upper lateral, and to replace it the dentist had devitalized the canine and inserted a platinum wire in the nerve canal. This wire, after being anchored by gutta-percha, was bent at right angles, and had a lateral soldered to it. No protection was given to the canine other than that afforded by the gutta-percha, so that the tooth soon decayed and gave way, the bar bent under the strain of mastication, and the lateral was forced up into the gum. Dr. Elliott removed the appliance, cut down the canine to near the gum, and fitted on a gold-backed plate tooth, with a hole through the gold for the passage of a screw which was anchored in the stump by amalgam. The lateral was soldered to the pivot thus made, and on the mesial side of the lateral a pin was soldered which passed into a small cavity already existing in the central, where it was secured by a filling. Subsequent experience proved the value of having the bridge detachable, for after the appliance had been worn for some months the pin in the central gave way; the bridge was then quickly removed by unscrewing the nut, a new pin soldered on, and the apparatus replaced.

Dr. Elliott considered that the attachment of an artifical crown by means of a screw and nut was decidedly the best mode of pivoting for these cases, on account of its being easily detachable in case of accident, This was almost impossible when bridge-work was attached to crowns fitted on the Richmond principle. He found also that it was very difficult to prevent food and mucus accumulating under the overlapping edges of these crowns and leading to bad results. His experienoe of this method of pivoting dated back some five or six years, the results at first being most discouraging. These failures taught him that in order to obtain satisfactory results he must make his own screws. He found that when he used those sold by the depots the nuts came unscrewed and the crowns came off, frequently in two or three weeks. The screws must be much finer than those usually sold, and the nuts must be conical and and cut half through, so as to make them self-locking. He found also that aluminium bronze or German silver were better materials for the screws than platinum, since they became slightly oxidized, and thus held more securely.

Dr. G. Field said he had not used bridge-work very extensively, but he found that in exceptional cases it answered admirably, and he used it under favorable conditions with great satisfaction to himself and to his patients. He had, of course, met with failures, but these had not been sufficient to discourage him, or to induce him to give up the method. He preferred the Richmond crown, or the Webb flat pivot tooth with gold backing, to the use of screws and nuts. In some cases he anchored the ends of the bar into adjoining natural teeth by means of gold fillings. The fillings would break away occasionally, this could not altogether be prevented, but as a rule they lasted very well. In one case where this happened, the patient being unwilling to submit to having another gold filling inserted, he filled with osteo; and although the filling had required attention from time to time to repair the effects of surface disintegration, it had lasted very well. He did not approve, as a rule, of the plan of bridging over four or five teeth, and had only seen one case in which this had been satisfactorily accomplished. The objection on the score of uncleanliness was entirely theoretical; the bridge could always be made of such a form as to be easily kept clean.

Dr. A. S. Richmond said the subject nnder dicussion, crown and bridge-work, was one to which he had given some attention, and which interested him greatly. Dr. W. M. Morison, of St. Louis, the inventor of the Morison chair and engine, was, he believed, the first to make gold crowns encircling the roots of molar and bicuspid teeth; this was in 1868. In 1876, Dr. Cassius M. Richmond, who was then practicing at San Francisco, produced the "Richmond crown." This was a porcelain, or porcelain-faced, crown attached to the natural root by a pin or tube, the attachment being strengthened by a gold band encircling the root. This method was still employed by the best operators throughout the world, and had never been improved upon since.

With regard to bridge-work he would hand round some models of cases which had been under his own care, showing various adaptations of the method. He had a piece of bridge-work in his own mouth which was put in in 1876, and which had therefore stood the test of nine years' wear. In 1878, he had a case in which a loose and decayed lateral adjoined a sound and healthy central. After trying various experiments with the lateral, he extracted it and attached a lateral crown to the central and canine: the work had stood well up to the present time. Early in 1880 he pivoted a first molar with a Morison gold crown and the canine on the same side with a porcelain-faced crown, connecting them by a bridge carrying the two bicuspids; this also was still in use. These cases might serve to show the lasting character of the work. There was no difficulty whatever in keeping it clean, the teeth being supported clear of the gum, and the whole fitted as it should be in a proper workmanlike manner.

Dr. W. Mitchell said he had had a case to deal with that day in which he had been obliged to modify the ordinary Richmond method. He had occasion to remove a Bonwill crown, but found the pin so firmly fixed in the root by amalgam that he found it impossible to remove it. He therefore cut it off even with the gum margin, and then with a fine fissure burr removed the amalgam all round it to the thickness of about two lines, and to the depth of about a quarter of an inch. He then made the band as usual, soldered on the top, and made a platina tube to fit the pin. He then cut away a portion of the top, placed the band and tube in position, and waxed them together in the mouth. They were then removed in their proper relation for soldering, after which he replaced them and fitted the tooth in the ordinary way. Then, after waxing together and investing in sand and plaster, the soldering was completed and the work was polished and inserted in the mouth, where it fitted perfectly. He thought this plan was really an improvement on the usual method, for the tube added stiffness to the structure, making it much firmer than where the ordinary pin alone was used. Where amalgam was used for setting the pin, of course platina both for pin and tube was indispensable, but he did not see why a good strong cement could not be used with advantage.

Mr. J. S. Turner said that whilst he could not help admiring the ingenuity displayed in these methods of pivoting and bridge-work, he was rather at a loss to know what was gained by all this elaboration. The results might be perfectly satisfactory in a certain number of cases, but it must be remembered that operators did not always know of their own failures. When patients were dissatisfied with what had been done for them by one practitioner, they were apt to go to another. He had lately come across a patient who had had five front teeth pivoted sixteen years ago, viz., two centrals, a lateral, and two canines, and they were still firm and useful. The pivoting was done in the old-fashioned way: the roots were cut down and polished, a pin inserted in the canal, and a model taken, then a tube tooth was fitted, first on the model and then to the root, a little floss silk being wound round the pin before it is forced into the root. The plan of pivoting with nuts and screws might sometimes be useful, but he thought that the insertion of the screw and tightening the nut must be a more unpleasant process than fitting a pin into the canal in the old way. He had seen many cases when with Mr. Cartwright of teeth which were shed in the ordinary course of nature with pivot-crowns attached which had been in use for a great number of years, and others must have frequently met with similar cases. Seeing then that the results of the old method were generally so satisfactory, he failed to see the advantage of these later and more elaborate methods.

Mr. R. H. Woodhouse said he had lately removed a pivoted tooth which had been in use for twenty-three years; the crown was quite firm, but the root was absorbed. He thought this was a triumph for the old method.

The Chairman said he had personally no experience of bridge-work, and he was disposed to agree with Mr. Turner that there was no great necessity for the use of screws and nuts. He had been in practice a good many years, and he thoroughly agreed with what Mr. Turner had said as to the good results obtained by the old method of pivoting stumps with natural teeth. The pins was made of *hard* gold; a little floss silk was wound round it, it was then moistened with mastic varnish, and forced well up the canal. Teeth pivoted in this way lasted from twelve to thirty years, and the pin never came out—no one ever thought of its doing so. He felt bound to admit, however, that he had not been quite so successful with mineral teeth. He would now call upon Dr. Elliott to reply.

Dr. Elliott said he had himself used the old method of pivoting, but he wished to advance with the times. The weak point of the method described by Mr. Turner was that the front edge of the tooth resting on the front of the stump gave a considerable amount of leverage, and as the result of any strain on the tooth the pivot was sometimes pulled out. Thus a patient of his who had a tooth pivoted in this way lost one crown and bent another. Another objection was that the pin would sometimes stop short in the canal, and use what force you might you could not get the crown close up to the stump.

In his lectures at the National Dental Hospital, he had been in the habit of describing sixty different methods of pivoting, but he only used two in his own practice, viz., the one he had already described and the Flagg process. This latter method he considered a very good one, and quite as simple as the older method; but though one of the best for front teeth it was not as well adapted to bicuspids as the other. A plain plate tooth was soldered to a pin; this was passed up the nerve canal and packed all about with amalgam. He had brought his drawer of pivoting instruments with him in case any of the members present might like to look over them.

The Chairman thanked Dr. Elliott, in the name of the Society, for his interesting communication, and also Messrs. Hunt, Lloyd Williams, Browne-Mason, Boyd Wallis, and other contributors of specimens and casual communications.

The Society then adjourned.—*The Dental Record.*

